# One-Pot Preparation of Imidazole-Ring-Modified Graphitic Carbon Nitride Nanozymes for Colorimetric Glucose Detection

**DOI:** 10.3390/bios12110930

**Published:** 2022-10-27

**Authors:** Yuanyuan Chen, Xueyou Gao, Hang Xue, Guohui Liu, Yue Zhou, Jian Peng

**Affiliations:** 1 Department of Pharmacology, Medical College, Wuhan University of Science and Technology, Wuhan 430022, China; 2 State Key Laboratory of Advanced Technology for Materials Synthesis and Processing, Biomedical Materials and Engineering Research Center of Hubei Province, Wuhan University of Technology, Wuhan 430070, China; 3 Department of Orthopaedics, Union Hospital, Tongji Medical College, Huazhong University of Science and Technology, Wuhan 430022, China

**Keywords:** nanozymes, biosensors, 2D materials, diabetic, colorimetric detection

## Abstract

Nanozymes are highly desired to overcome the shortcomings of natural enzymes, such as low stability, high cost and difficult storage during biosensing applications. Herein, by imitating the structure of natural enzymes, we propose a one-pot annealing process to synthesis imidazole-ring-modified graphitic carbon nitride (*g*-C_3_N_4_-Im) with enhanced peroxidase-like activity. *g*-C_3_N_4_-Im shows enhanced peroxidase-like activity by 46.5 times compared to unmodified *g*-C_3_N_4_. Furthermore, imidazole rings of *g*-C_3_N_4_-Im make it possible to anchor Cu(II) active sites on it to produce *g*-C_3_N_4_-Im-Cu, which shows a further increase in peroxidase-like activity by three times. It should be noted that the as-prepared *g*-C_3_N_4_-Im-Cu could show obvious peroxidase-like activity over a broad range of pH values and at a low temperature (5 °C). The ultrahigh peroxidase-like activity is attributed to the electronic effect of imidazole rings and the active sites of Cu(II) for ·OH production. Based on the enhanced peroxidase-like activity, a H_2_O_2_ and glucose biosensor was developed with a high sensitivity (limit of detection, 10 nM) and selectivity. Therefore, the biosensor shows potential for applications in diabetic diagnoses in clinical practice.

## 1. Introduction

Natural enzymes have been widely used in industrial, medical, environmental and biosensing applications, owing to their high catalytic activity and substrate specificity. However, the high-cost processing, difficulties of recycling and poor stability limit its applications. Compared with natural enzymes, nanozymes exhibit significant advantages, such as low cost, adjustable catalytic activities and flexibility in structure design, which make them gradually become substitutes for natural enzymes and be gradually applied to lots of fields, especially in biosensing [1,2,3,4,5,6,7,8]. Because Fe_3_O_4_ nanoparticles were first reported in 2007 to show intrinsic peroxidase-like activity by Yan’s group [9], lots of nanozymes, such as noble metal [10,11,12,13,14], metallic oxide [15,16], metal sulfide [17,18], graphene oxide [19,20,21], and carbon quantum dots [22,23], have been reported. Among them, two-dimensional (2D) materials, such as GO, have been widely explored as nanozymes, owing to its large specific surface area and high atomic utilization [24,25]. Although GO-based materials have been widely explored as efficient nanozymes, the development of a two-dimensional artificial enzyme with a low cost and by a simple and convenient method is still highly desired.

Graphite-phase carbon nitride (*g*-C_3_N_4_) is the most stable allotrope of carbon nitride under ambient conditions [26,27,28]. *g*-C_3_N_4_ consists of carbon and nitrogen, which are abundant elements, and is relatively environmentally friendly, sustainable and can be produced on a large scale with low costs. Especially, *g*-C_3_N_4_ can be used for the detection of H_2_O_2_ and glucose because of its intrinsic peroxidase-like activity [29]. However, the catalytic activity of *g*-C_3_N_4_ is far from satisfactory, compared with natural peroxidase. As such, it is essential to design highly efficient *g*-C_3_N_4_-based nanozymes. In previous reports, many strategies, such as composites, element doping, and modifications, have been proposed to enhance the activity of *g*-C_3_N_4_ [30,31]. However, it still remains a challenge to prepare *g*-C_3_N_4_-based nanozymes with high catalytic activity in a wide pH range and at a low temperature; thus, they could be used for the sensitive detection of glucose in human urine.

**Figure 1:**
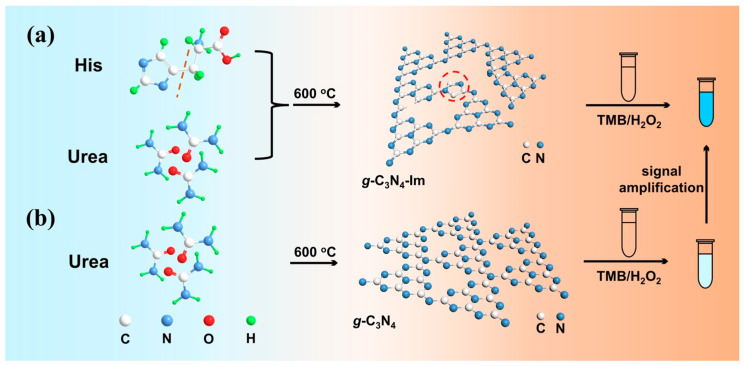
Schematic illustration of one-pot annealing preparation of *g*-C_3_N_4_-Im (**a**) and *g*-C_3_N_4_ (**b**) with enhanced peroxidase-like activity for H_2_O_2_ detection.

Herein, by imitating the structure of natural enzymes [9,32,33], a one-pot annealing process was proposed to construct imidazole-ring-modified graphitic carbon nitride (*g*-C_3_N_4_-Im) with enhanced peroxidase-like activity (Figure 1). In this system, histidine was added during calcination to produce *g*-C_3_N_4_-Im, showing enhanced peroxidase-like activity by 46.5 times compared to unmodified *g*-C_3_N_4_. Furthermore, imidazole rings of *g*-C_3_N_4_-Im make it possible to anchor Cu(II) active sites onto it to produce *g*-C_3_N_4_-Im-Cu, which shows a further increase in peroxidase-like activity by three times. It was demonstrated that the *g*-C_3_N_4_-Im-Cu nanozyme could work efficiently in wide pH values (4–9) and at a low temperature (5 °C). The ultrahigh peroxidase-like activity was attributed to the electronic effect of imidazole rings and the active sites of Cu(II) for ·OH production. Based on the enhanced peroxidase-like activity, a H_2_O_2_ and glucose biosensor was developed with high sensitivity and selectivity. *g*-C_3_N_4_-Im-Cu was further applied to detect glucose in human urine samples with high sensitivity and selectivity.

## 2. Experimental

### 2.1. Reagents and Materials

The purity of reagent was analytically pure. Copper (II) acetate monohydrate and histidine were obtained from Aladdin Reagent Co., Ltd. (Shanghai, China). Urea, a 30 wt% H_2_O_2_ solution, terephthalic acid (TA) and glucose were purchased from Sinopharm Chemical Reagent Co., Ltd. (Shanghai, China). 3,3′,5,5′-tetramethylbenzidine (TMB), methylene blue (MB), 2,2′-azino-bis(3-ethylbenzothiazoline-6-sulfonic acid) (ABTS) and o-phenylenediamine (OPD) were a product of J&K Chemical Reagent Ltd. (Shanghai, China). Glucose oxidase (GO_x_) was purchased from Sigma-Aldrich Reagent Ltd. (Burlington, MA, USA). Urine samples of diabetic patients and healthy controls (20 samples) were obtained from the Union Hospital, Tongji Medical College, Huazhong University of Science and Technology.

### 2.2. Preparation of g-C_3_N_4_-Im and g-C_3_N_4_-Im-Cu Nanosheets

The preparation of *g*-C_3_N_4_ was according to the previous literature [34,35]. The preparation of *g*-C_3_N_4_-Im was as follows: firstly, 0.77g of histidine was mixed with 30 g of urea under grinding. Then, the mixture was transferred to an alumina crucible followed, by heating in a muffle furnace from room temperature to 600 °C with a heating rate of 5 °C min^−1^, followed by calcination at 600 °C for 4 h. Finally, *g*-C_3_N_4_-Im was obtained after natural cooling. *g*-C_3_N_4_-Im of different mole ratios can be obtained by changing the mass ratio of urea to histidine. The preparation of *g*-C_3_N_4_-Im-Cu was obtained by mixing copper (II) acetate (2 mM) and *g*-C_3_N_4_-Im (0.5 mg/mL) suspension in equal volumes under stirring. This stable *g*-C_3_N_4_-Im-Cu suspension was dialyzed overnight to remove free Cu(II) ions and other byproducts, and the final Cu(II) in *g*-C_3_N_4_-Im-Cu was diluted by 100 times before being measured by the inductively coupled plasma mass spectrometry (ICP-MS) technique and the energy-dispersive X-ray (EDX) spectrum.

### 2.3. Instruments and Characterization

The crystal structure was characterized by X-ray diffraction (XRD, Bruker D8 advance, United States) at 40 kV and 40 mA. The morphology of the samples was observed by a scanning electron microscope at 10 kV (SEM, Hitachi SU8010, Tokyo, Japan). FTIR spectra (Bruker Alpha II, Billerica, MA, USA) and Raman spectra (Thermo Fischer DXR, Waltham, MA, USA) were used to characterize the molecular structure of the samples. X-ray photoelectron spectroscopy (XPS, Thermo ESCALAB 250XI, Waltham, MA, USA) was used to characterize the fine structure of the products. For thermogravimetric analysis, 20 mg of the dry sample was sealed, with the heating temperature increasing from room temperature to 1000 °C at a heating rate of 10 °C·min^−1^ in nitrogen atmosphere.

### 2.4. Peroxidase-like Activity Assays of g-C_3_N_4_-Im-Cu Nanosheets

The peroxidase-like activity of *g*-C_3_N_4_-Im-Cu nanosheets was evaluated as follows: 500 μL of *g*-C_3_N_4_-Im-Cu suspension, 500 μL of H_2_O_2_ (100 mM), 500 μL of TMB (2 mM) and 1.5 mL of NaAc/Hac buffer (10 mM, pH = 7) were mixed in a cuvette before the UV–Vis spectra were collected from 400~800 nm. The assays were monitored in wavelength-scan mode or in time-dependent mode at 652 nm under the optimal conditions as described above, unless otherwise stated.

### 2.5. Detection of H_2_O_2_ and Glucose

For the detection of H_2_O_2_, 500 μL *g*-C_3_N_4_-Im-Cu, a 500 μL TMB (6 mM) solution and a 1.5 mL NaAc/HAc buffer (10 mM, pH = 7) were added to a 500 μL H_2_O_2_ solution of different concentrations. The absorbance of the mixed solution at 652 nm was recorded with a UV–Vis–NIR spectrometer (Shimadzu UV-1900i, Kyoto, Japan).

Glucose detection methods were carried out as follows: firstly, 60 ul GO_x_ solution (10 mg/mL) with a 540 μL glucose solution of different concentrations were incubated at 37 °C for 40 min. Then, 10 μL H_2_SO_4_ (10%, *v*/*v*) was added into the mixture to stop the reaction of glucose oxidation. Finally, 500 μL *g*-C_3_N_4_-Im-Cu, a 500 μL TMB (6 mM) solution and a 1.5 mL NaAc/HAc buffer (10 mM, pH = 7) were added to the above glucose reaction solution, and the absorbance (652 nm) of the mixture was collected at 15 °C.

### 2.6. Clinical Samples Analysis

The analysis of urine samples of diabetic patients and healthy controls was performed as follows: 540 μL of urine was mixed with a 60 μL glucose oxidase solution (10 mg/mL), followed by incubation at 37 °C for 40 min. Then, the mixture was mixed with 10 μL H_2_SO_4_ (10%, *v*/*v*) to stop reaction. Finally, the reaction mixture was centrifuged at 8000 rpm for 10 min to remove the precipitate and the supernatant was collected (500 μL) and mixed with 500 μL *g*-C_3_N_4_-Im-Cu, a 500 μL TMB (6 mM) solution and 1.5 mL NaAc/HAc buffer (10 mM, pH = 7), before the absorbance at 652 nm was recorded.

## 3. Results

**Figure 2:**
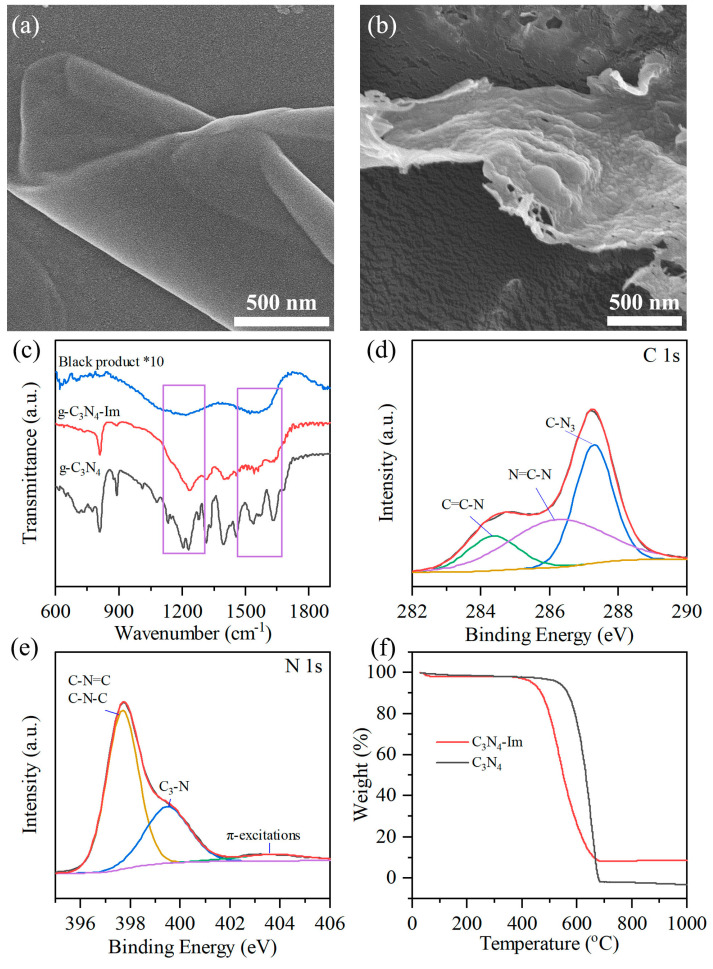
SEM image of (**a**) *g*-C_3_N_4_ and (**b**) *g*-C_3_N_4_-Im. (**c**) FTIR spectra of *g*-C_3_N_4_, *g*-C_3_N_4_-Im and annealed histidine. High-resolution (**d**) C_1s_ and (**e**) N_1s_ XPS spectra of *g*-C_3_N_4_-Im. (**f**) TGA curves of *g*-C_3_N_4_ and *g*-C_3_N_4_-Im.

The structure of *g*-C_3_N_4_-Im was characterized by X-ray diffraction (XRD). As shown in Figure S1 (Supplementary Materials), the two typical peaks of *g*-C_3_N_4_-Im at 12.8° and 27.1° exhibited a lower intensity than *g*-C_3_N_4_ [36]; meanwhile, the peak at 27.1° was shifted to a low degree compared with *g*-C_3_N_4_, which indicates that the ordered triazine structure was partly destructed [37]. The morphology and structure of *g*-C_3_N_4_ and *g*-C_3_N_4_-Im were observed by SEM (Figure 2a,b). The structure of *g*-C_3_N_4_ comprised ultrathin two-dimensional nanosheets of a large size (>1 µm), which is the typical structure of the graphitic phase (Figure 2a). In contrast, there were many folds and holes for the structure of *g*-C_3_N_4_-Im (Figure 2b), which further confirms the results from XRD. To figure out the chemical structure of *g*-C_3_N_4_-Im, FTIR data were collected (Figure 2c). The spectra of *g*-C_3_N_4_-Im showed two broad peaks, ranging from 1050 cm^−1^–1350 cm^−1^ and 1420 cm^−1^–1630 cm^−1^, respectively, which are attributed to the imidazole ring (Figure S2) [38,39,40,41]. This result was further confirmed by Raman spectra (Figure S3). The broad peaks, ranging from 1000 cm^−1^–1750 cm^−1^, are ascribed to superposition peak *g*-C_3_N_4_-Im and imidazole [42].

To further investigate the fine chemical structure of *g*-C_3_N_4_-Im, X-ray photoelectron spectroscopy (XPS) was performed. As shown in Figure 2d, the peaks with binding energies of 284.4 eV, 286.3 eV and 287.3 eV belong to N-C=(-C) on the imidazole ring, N-C=N(-C) and C-N_3_ on *g*-C_3_N_4_, respectively [43]. For the high-resolution spectra of N_1s_ (Figure 2e), the typical peaks at 379.6 eV and 399.4 eV are attributed to C-N-C and N-C_3_, respectively. The weak peak at 403.6 eV is attributed to the π excitation [44]. The above results indicate that *g*-C_3_N_4_-Im was successfully prepared. To figure out the content of imidazole in *g*-C_3_N_4_-Im, TGA analysis was performed. As shown in Figure 2f and Figure S4, with the temperatures increase from 520 °C to 680 °C, *g*-C_3_N_4_ decreased rapidly and finally decomposed totally [45]. In contrast, *g*-C_3_N_4_-Im started to decompose at 410 °C, decomposed totally at 680 °C, and then 8 wt% of the carbide residue was collected. We found that there were 17 wt% imidazole in the *g*-C_3_N_4_-Im samples.

**Figure 3:**
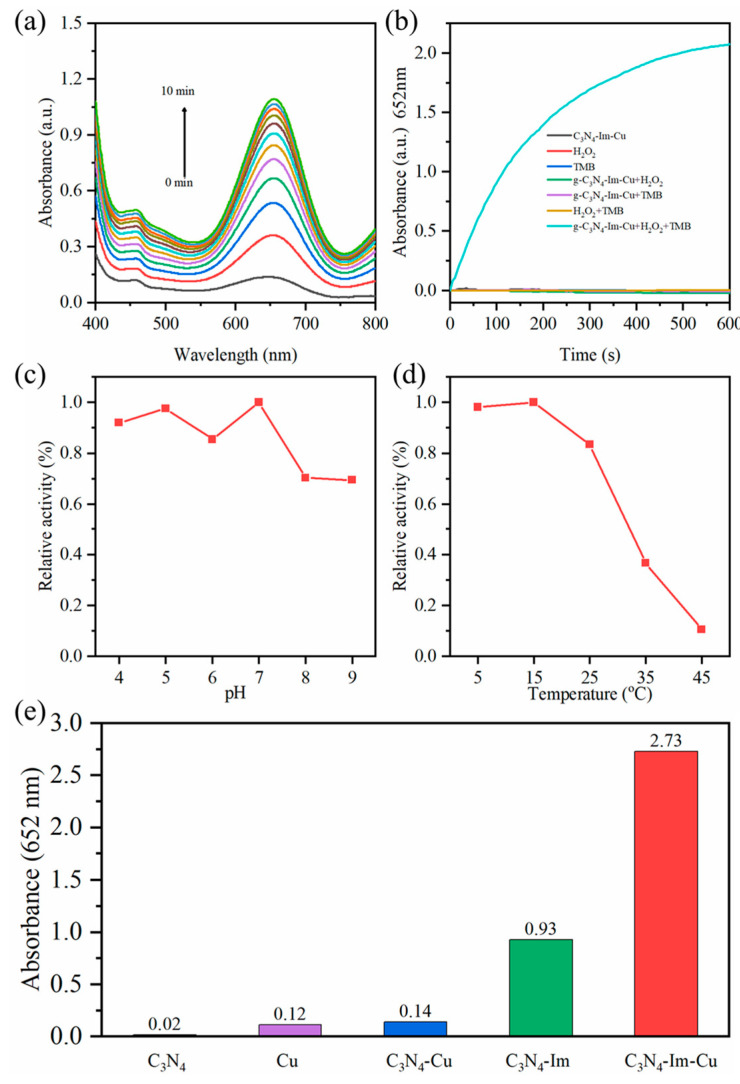
(**a**) UV–Vis spectra of mixed solution of TMB, H_2_O_2_ and *g*-C_3_N_4_-Im-Cu with time increasing. (**b**) The time-dependent absorbance changes at 652 nm of different catalysts. The determining factors in the test of peroxidase-like activity of *g*-C_3_N_4_-Im-Cu were (**c**) temperature and (**d**) pH. (**e**) The absorbance value of 652 nm at 10 min of TMB and H_2_O_2_ solution in the presence of *g*-C_3_N_4_ (black), Cu(Ac)_2_ (purple), *g*-C_3_N_4_-Cu (blue), *g*-C_3_N_4_-Im (green) and *g*-C_3_N_4_-Im-Cu (red).

To evaluate the peroxidase-like activity of *g*-C_3_N_4_-Im, the TMB-H_2_O_2_ reaction was used as model reaction because the colorless TMB can be oxidized by H_2_O_2_ to a blue product in the presence of a nanozyme. The catalytic activity of *g*-C_3_N_4_-Im was firstly evaluated by recording the spectra from 200–800 nm with a UV–Vis spectrometer (Figure 3a). Figure S5d shows the peroxidase-like activity comparison of *g*-C_3_N_4_, histidine and *g*-C_3_N_4_-Im of different concentrations, indicating that the preparation conditions of *g*-C_3_N_4_-Im can be optimized to be 100 urea: 1 histidine, which exhibited the highest peroxidase-like activity among all the samples. Therefore, a 100 urea: 1 histidine-derived *g*-C_3_N_4_-Im sample was used in the following experiments, unless otherwise stated.

To further enhance the catalytic activity of *g*-C_3_N_4_-Im, a Cu(II) ion was introduced into the suspension of *g*-C_3_N_4_-Im to produce *g*-C_3_N_4_-Im-Cu (see details in Experimental Section 2.2). EDS mapping indicated that Cu(II) was uniformly dispersed on the *g*-C_3_N_4_-Im-Cu nanosheets, and the Cu(II) content in *g*-C_3_N_4_-Im-Cu was measured to be 3.7 at% (Figures S5 and S6). The added concentration of Cu(II) was optimized to be 1 mM (Figure S7a). As expected, upon Cu(II) ion adsorption, *g*-C_3_N_4_-Im-Cu obviously showed three-times-enhanced catalytic activity (Figure S7e). To further confirm the peroxidase-like activity of *g*-C_3_N_4_-Im-Cu, ABTS and OPD were used as a substitute for TMB (Figure S8a–c). All results indicated that *g*-C_3_N_4_-Im-Cu shows an obvious peroxidase-like activity and that the TMB-H_2_O_2_ reaction was the most suitable reaction (Figure S8d). Figure 3b demonstrates that both H_2_O_2_ and *g*-C_3_N_4_-Im-Cu alone cannot oxidize TMB efficiently to produce a blue color. Consequently, TMB oxidation resulted from the decomposition of H_2_O_2_ by *g*-C_3_N_4_-Im-Cu. Figure 3c demonstrates that *g*-C_3_N_4_-Im-Cu showed intrinsic peroxidase-like activity in wide pH values from 4 to 9. Unexpectedly, *g*-C_3_N_4_-Im-Cu showed obvious catalytic activity, even under very low temperatures (<5 °C, Figure 3d). The above results indicate that *g*-C_3_N_4_-Im-Cu showed outstanding peroxidase-like activity, breaking the limitations of temperature and pH conditions.

To quantify the enhancement factor of catalytic activity for *g*-C_3_N_4_ before and after imidazole modification, the peroxidase-like activity of *g*-C_3_N_4_, Cu(II), *g*-C_3_N_4_-Cu, *g*-C_3_N_4_-Im and *g*-C_3_N_4_-Im-Cu are evaluated in Figure 3e. It was indicated that the peroxidase-like activity of *g*-C_3_N_4_ can be increased by 46.5 times after the imidazole modification, which is much better than bare *g*-C_3_N_4_ and Cu(II) under the same concentration. Furthermore, the peroxidase-like activity of *g*-C_3_N_4_-Im could be further enhanced a further three times after a simple Cu(II) coordination. Thus, the catalytic activity of *g*-C_3_N_4_ could be enhanced by over two orders of magnitude by a simple modification.

To probe the enhanced mechanism of catalytic activity of *g*-C_3_N_4_-Im-Cu, the thermodynamic parameters were measured. As shown in Figure S7f, the thermodynamic of H_2_O_2_ oxidation catalyzed by *g*-C_3_N_4_-Im-Cu was investigated under different temperatures (5 °C, 15 °C and 25 °C). The activation energy ($E_{a}$) of *g*-C_3_N_4_-Im-Cu was 11.54 kJ·mol^−1^ (Table S1) [46,47,48,49,50], which was much lower than that of other reported catalysts (Table S2), helping to understand the mechanisms of the high catalytic activity of *g*-C_3_N_4_-Im-Cu from the viewpoint of thermodynamics. To acquire kinetic parameters, enzyme kinetic experiments of *g*-C_3_N_4_-Im-Cu, with H_2_O_2_ and TMB as substrates, were performed. The optimal pH, temperature, H_2_O_2_ concentration and TMB concentration were obtained from Figure 3c,d and Figure S7b,c. Then, a series of kinetic experiments were carried out by adjusting the concentration of one substrate and fixing the concentration of another (Figure S9a,b). The Michaelis–Menten curve and Lineweaver–Burk curve (Figure S9c–f) were plotted, and the kinetic parameters $K_{m}$ and $V_{max}$ can be obtained from Table 1. In contrast to other reports, the $K_{m}$ value of *g*-C_3_N_4_-Im-Cu reached 0.034 mM^−1^ for TMB and 0.022 mM^−1^ for H_2_O_2_, which is much lower than that of FeS [51] and HPR [9]. The V_max_ value of *g*-C_3_N_4_-Im-Cu for H_2_O_2_ and TMB were much higher than that of HPR, respectively. Therefore, *g*-C_3_N_4_-Im-Cu exhibited a higher catalytic activity than that of natural enzyme HRP.

**Figure 4:**
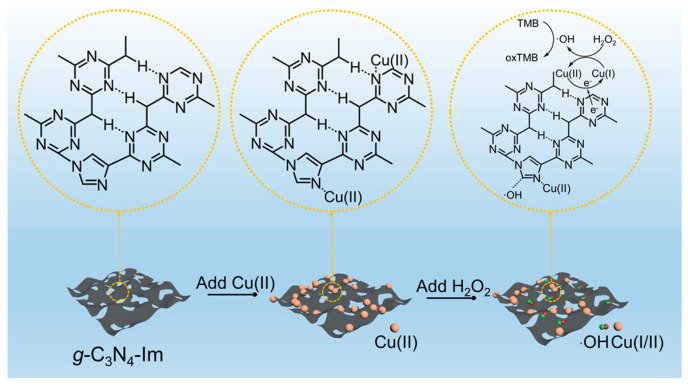
Suggested catalytic mechanism of *g*-C_3_N_4_-Im-Cu for H_2_O_2_ decomposition.

To further investigate the catalytic mechanisms of TMB oxidation, radical trapping experiments were performed. The decreased absorbance value and enhanced fluorescence signal in Figure S10 indicated that ·OH radicals were produced during the catalytic reactions [48]. Based on the above experiments, the suggested catalytic mechanism was proposed (Figure 4 and Figure S11). The intercalation of the imidazole ring broke the ordered structure of *g*-C_3_N_4_-Im and, upon adding Cu(II), the Cu(II) was captured by the imidazole ring and C-N heterocycle as active sites. In the reaction of catalyzed TMB oxidation, Cu(II) ions can be reversibly converted between Cu(II) and Cu(I) in H_2_O_2_ solutions [52,53]; meanwhile, ·OH could be stabilized on the imidazole ring, thus accelerating the decomposition of hydrogen peroxide.

**Figure 5:**
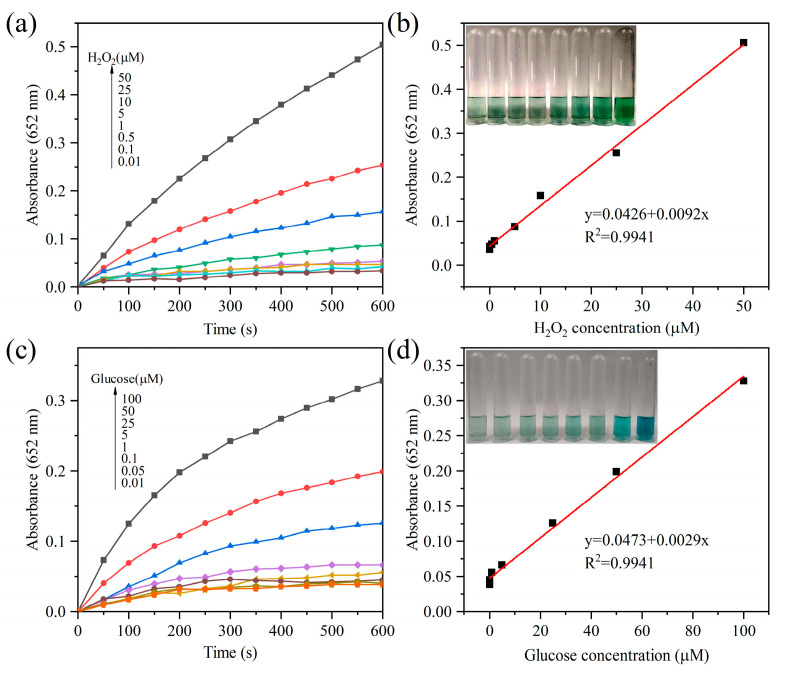
The time-dependent absorbance changes at 652 nm with different concentrations of (**a**) H_2_O_2_ and (**c**) Glucose. Linear calibration plot for (**b**) H_2_O_2_ and (**d**) glucose, calculated from (**a**) and (**c**), respectively. Inset: optical photograph of colored products in H_2_O_2_ and glucose with different concentrations.

Owing to the outstanding peroxidase-like activity of *g*-C_3_N_4_-Im-Cu, a simple colorimetric method was developed to detect H_2_O_2_. Figure 5a shows the response curve of H_2_O_2_ concentration to the absorbance of oxTMB. It can be observed from Figure 5b that a linear relationship (*y* = 0.0092 × *x* + 0.0426) was established in the range of 0.01~50 mM, with a correlation coefficient of 0.9941 and a detection limit of H_2_O_2_ of 10 nM (the signal-to-noise ratio was two). As H_2_O_2_ is the product of the GO_x_-catalyzed oxidation of glucose when combined with GO_x_ (Figure S9), the proposed colorimetric strategy was developed for the detection of glucose. As shown in Figure 5c, the response curve of glucose indicated that the linear relationship (*y* = 0.0029*x + 0.0473) of glucose concentration with the absorbance of oxTMB ranged from 0.01 to 100 mM (R^2^ = 0.9941), and the detection limit was 10 nM (the signal-to-noise ratio was two, Figure 5d and Table S3), which is more sensitive than that of many other reports [31,58,59,60,61,62,63,64,65,66,67,68,69,70,71,72,73,74,75,76,77,78,79,80,81,82,83,84,85,86,87,88]. These results indicate that *g*-C_3_N_4_-Im-Cu exhibits good sensitivity for glucose detection.

**Figure 6:**
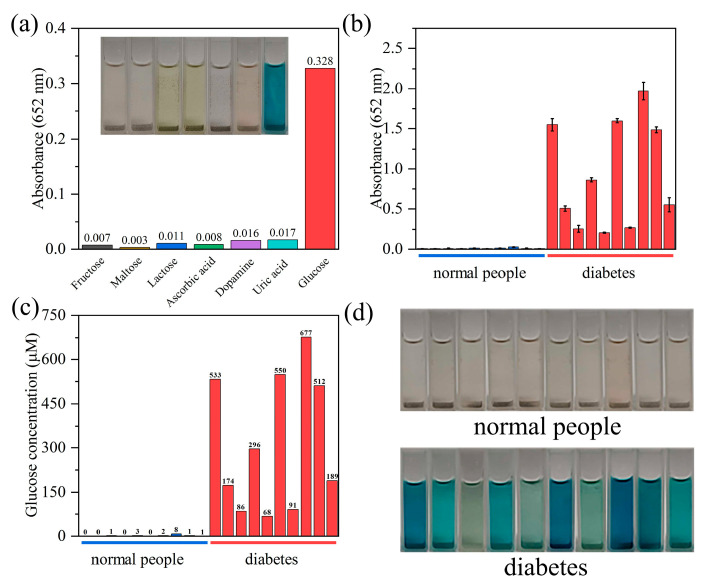
(**a**) Selective detection of glucose. Insert: Optical photographs of oxidized products in 0.1 mM fructose, maltose, lactose, ascorbic acid, dopamine, uric acid and glucose. (**b**) Detection of glucose of human urine of diabetic patients and healthy controls. (**c**) Glucose concentration in the 6.8 times diluted urine samples. (**d**) Optical photographs of oxidized products in human urine.

To explore the possibility of the use of biosensor for glucose detection in real samples, lots of experiments were performed as follows: firstly, fructose, maltose, lactose, ascorbic acid, dopamine and uric acid were used to investigate the selectivity and anti-interference of this biosensor. Figure 6a demonstrates that the absorbance of oxidized products from these glucose analogs and interfering substances were negligible in contrast to that of glucose under the same concentration, indicating that this biosensor shows a satisfactory selectivity and anti-interference for glucose, which resulted from the high affinity of glucose oxidase for glucose. Meanwhile, 20 samples of clinical urine samples of diabetic patients and healthy controls obtained from Union Hospital, Tongji Medical College were used for the following analysis. As shown in Figure 6b–d, the absorbance (652 nm) of urine samples from diabetic patients behaved over two orders of magnitude higher than that of the healthy controls. Thus, we can easily distinguish the urine samples of normal people (no color change) and diabetic patients (blue color) by the naked eye (Figure 6d). Consequently, the results indicate that the *g*-C_3_N_4_-Im-Cu-based biosensor shows potential for applications in the detection of glucose from clinical urine samples.

## 4. Conclusions

In summary, a one-pot annealing process to prepare *g*-C_3_N_4_-Im with enhanced peroxidase-like activity was demonstrated. Upon Cu(II) coordination, *g*-C_3_N_4_-Im-Cu showed 139.5 times increased peroxidase-like activity compared to unmodified *g*-C_3_N_4_. Interestingly, the as-prepared *g*-C_3_N_4_-Im-Cu could work in wide pH values (4–9) and at a low temperature (5 °C). Then, a simple, sensitive and selective colorimetric biosensor was constructed to sensitively detect H_2_O_2_ and glucose. Importantly, the biosensor was used to determinate clinical urine samples of diabetes with satisfactory results. *g*-C_3_N_4_-Im-Cu shows several advantages over natural enzymes and other existing alternatives, such as easy preparation, low cost, high performance at low temperatures and a universal pH, thus showing the potential applications in medical diagnostics, environmental monitoring and catalysis.

## Figures and Tables

**Table 1:** Kinetic parameters for TMB oxidation catalyzed by different catalysts.

Catalyst	K_m_ (mM^−1^)		V_max_ (10^−8^ M·s^−1^)	
	TMB	H_2_O_2_	TMB	H_2_O_2_
*g*-C_3_N_4_-Im-Cu	0.034	0.022	42.05	45.16
Ag@Fabric [54]	0.19	7.61	15.10	14.40
Fe_3_O_4_@MIL-100(Fe) [55]	0.112	0.077	11.42	17.95
BNNS@CuS [56]	0.175	25	3.76	12.5
HRP [57]	0.43	3.70	10.00	8.71

## Data Availability

Not applicable.

## Supplementary Materials

The following supporting information can be downloaded at: https://www.mdpi.com/article/10.3390/bios12110930/s1, Figure S1: XRD pattern of histidine, *g*-C_3_N_4_, and *g*-C_3_N_4_-Im of different mass ratios between urea and histidine (10:1; 100:1; 1000:1) after calcination; Figure S2: (a) FTIR spectra of *g*-C_3_N_4_ before and after calcination; (b) FTIR spectra of histidine and *g*-C_3_N_4_-Im of different mass ratios between urea and histidine (10:1; 100:1; 1000:1) after calcination; Figure S3: Raman spectra of heating the *g*-C_3_N_4_ and the *g*-C_3_N_4_-Im; Figure S4: DTG curve of *g*-C_3_N_4_ and *g*-C_3_N_4_-Im; Figure S5: SEM image (a) and corresponding EDS mapping images (b–d) of *g*-C_3_N_4_-Im-Cu nanosheets; Figure S6: EDX spectrum of *g*-C_3_N_4_-Im-Cu nanosheets showing the content of C, N, Cu and O elements; Figure S7: Optimal reaction conditions for peroxidase-like activity of *g*-C_3_N_4_-Im-Cu; Figure S8: Peroxidase-like activity of *g*-C_3_N_4_-Im-Cu with various substrates; Figure S9: Steady-state kinetic assay of peroxidase-like activity of *g*-C_3_N_4_-Im-Cu; Figure S10: Detecting hydroxyl radical (•OH) with UV-vis and fluorescence spectra; Figure S11: Schematic illustration of glucose detection with glucose oxidase (GOx) and *g*-C_3_N_4_-Im-Cu catalyzed reactions. Table S1: Activation energy calculation results; Table S2: Activation energy for H_2_O_2_ oxidation catalyzed by different catalysts; Table S3: Limit of detection (LOD) of various nanozymes for glucose. References [31,46,47,48,49,50,58,59,60,61,62,63,64,65,66,67,68,69,70,71,72,73,74,75,76,77,78,79,80,81,82,83,84,85] are cited in the supplementary materials.
