# Supplementary material for: One-Pot Preparation of Imidazole-Ring-Modified Graphitic Carbon Nitride Nanozymes for Colorimetric Glucose Detection
Source: Biosensors (Basel). 2022 Oct 27;12(11):930. doi: 10.3390/bios12110930 (PMC9688121; doi:10.3390/bios12110930)
Supplement: Supplementary file 1 [file biosensors-12-00930-s001.zip › biosensors-1921441-supplementary.pdf]

Supplementary Materials

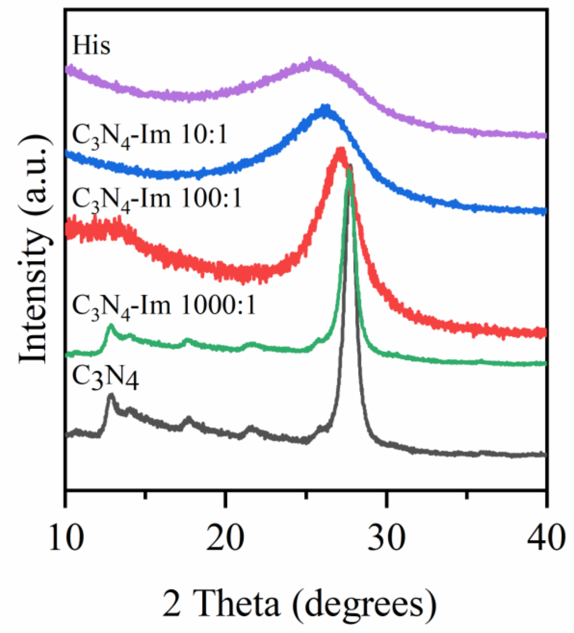

Figure S1. XRD pattern of histidine, g-C_3N_4, and g-C_3N_4-Im of different mass ratios between urea and histidine (10:1; 100:1; 1000:1) after calcination.

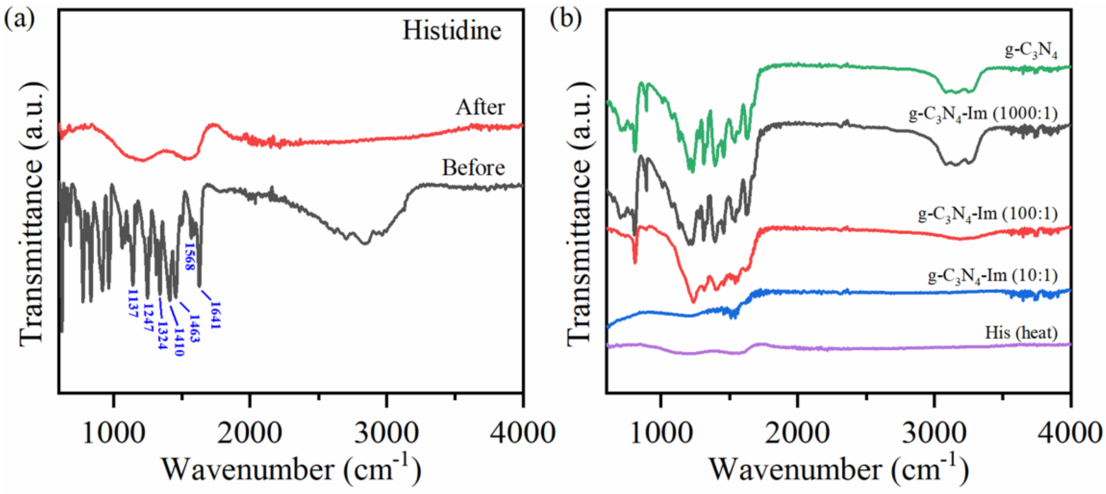

Figure S2. a) FTIR spectra of g-C_3N_4 before and after calcination; b) FTIR spectra of histidine and g-C_3N_4-Im of different mass ratios between urea and histidine (10:1; 100:1; 1000:1) after calcination.

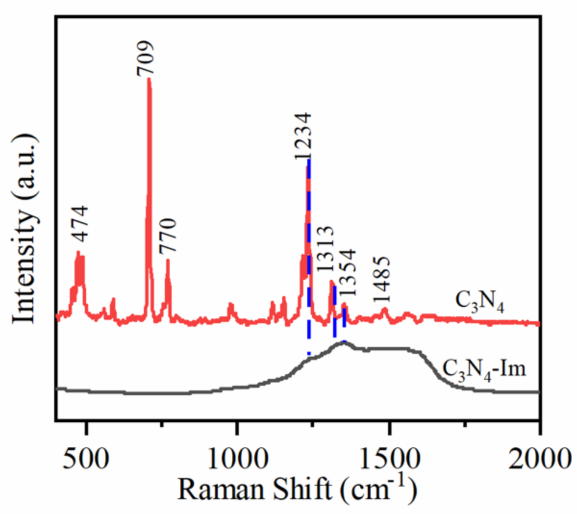

Figure S3. Raman spectra of heating the g-C_3N_4 and the g-C_3N_4-Im.

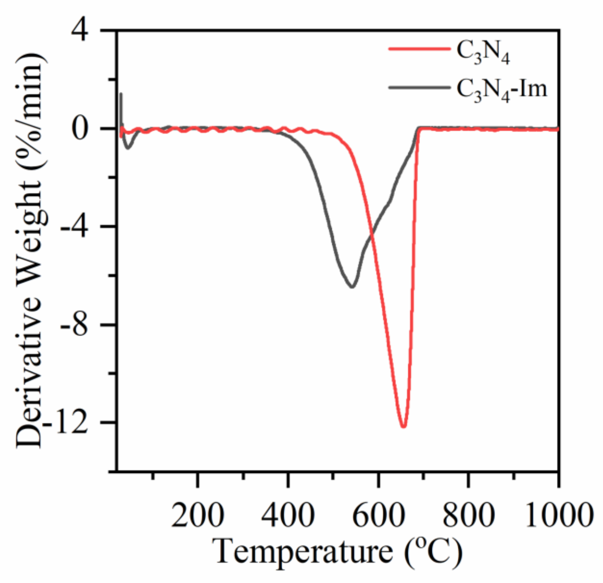

Figure S4. DTG curve of g-C_3N_4 and g-C_3N_4-Im.

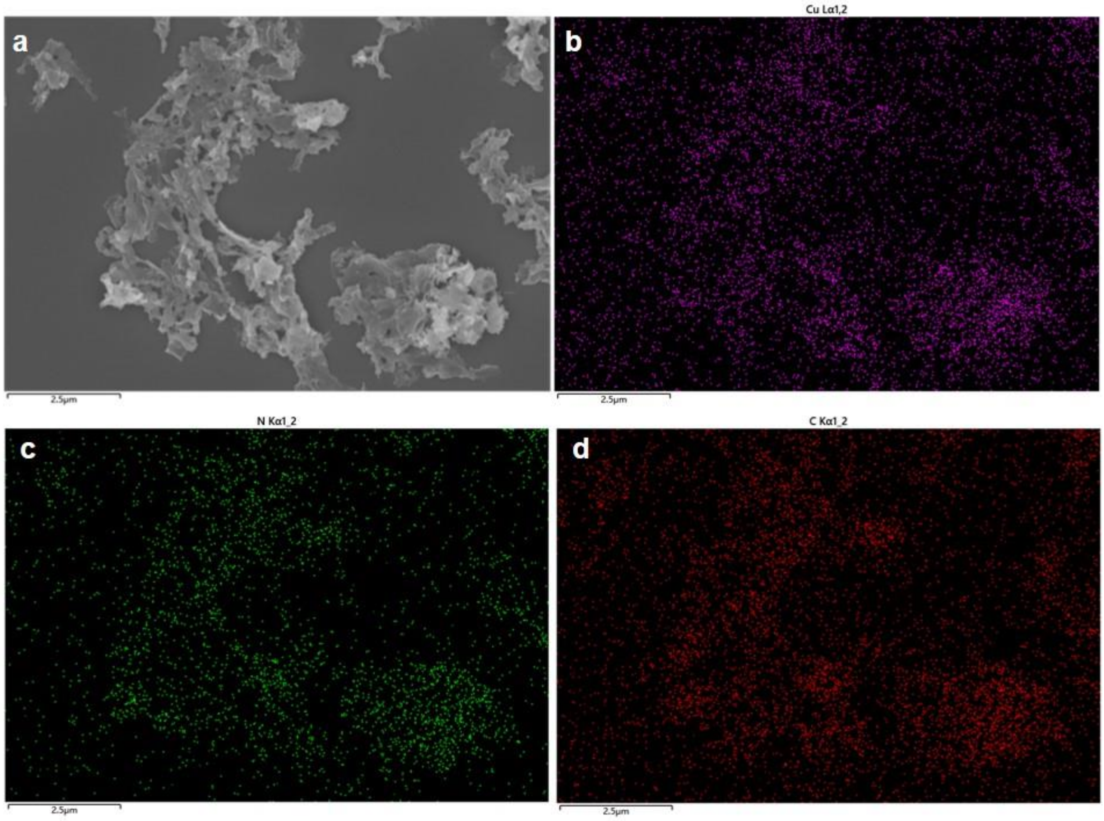

Figure S5. SEM image (a) and corresponding EDS mapping images (b-d) of g-C_3N_4-Im-Cu nanosheets.

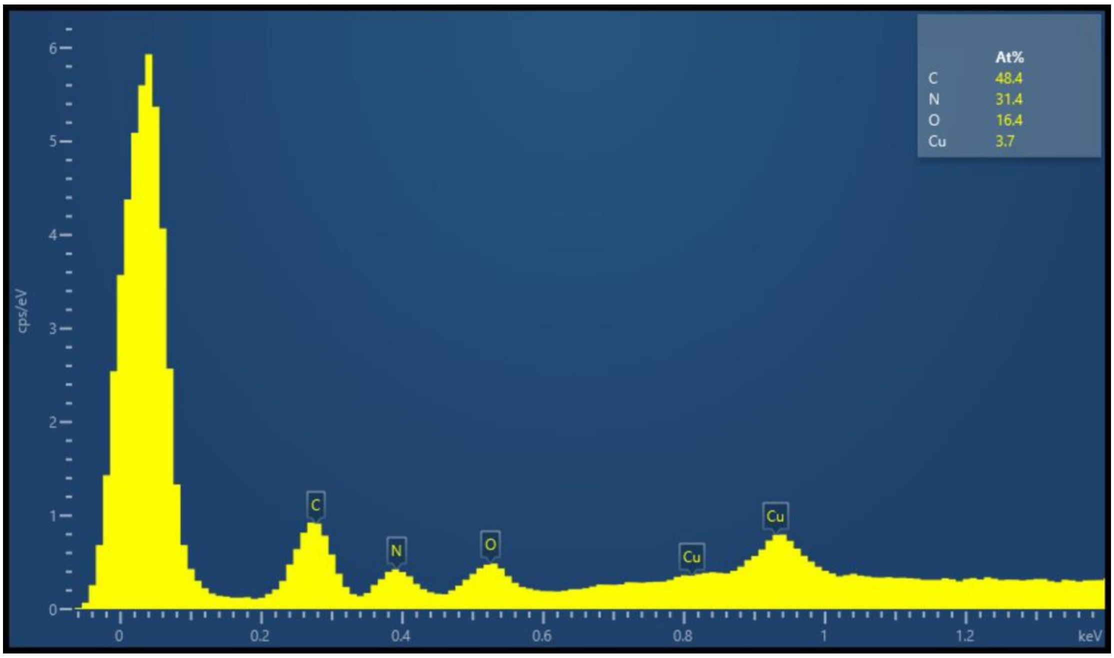

Figure S6. EDX spectrum of g-C_3N_4-Im-Cu nanosheets showing the content of C, N, Cu and O elements.

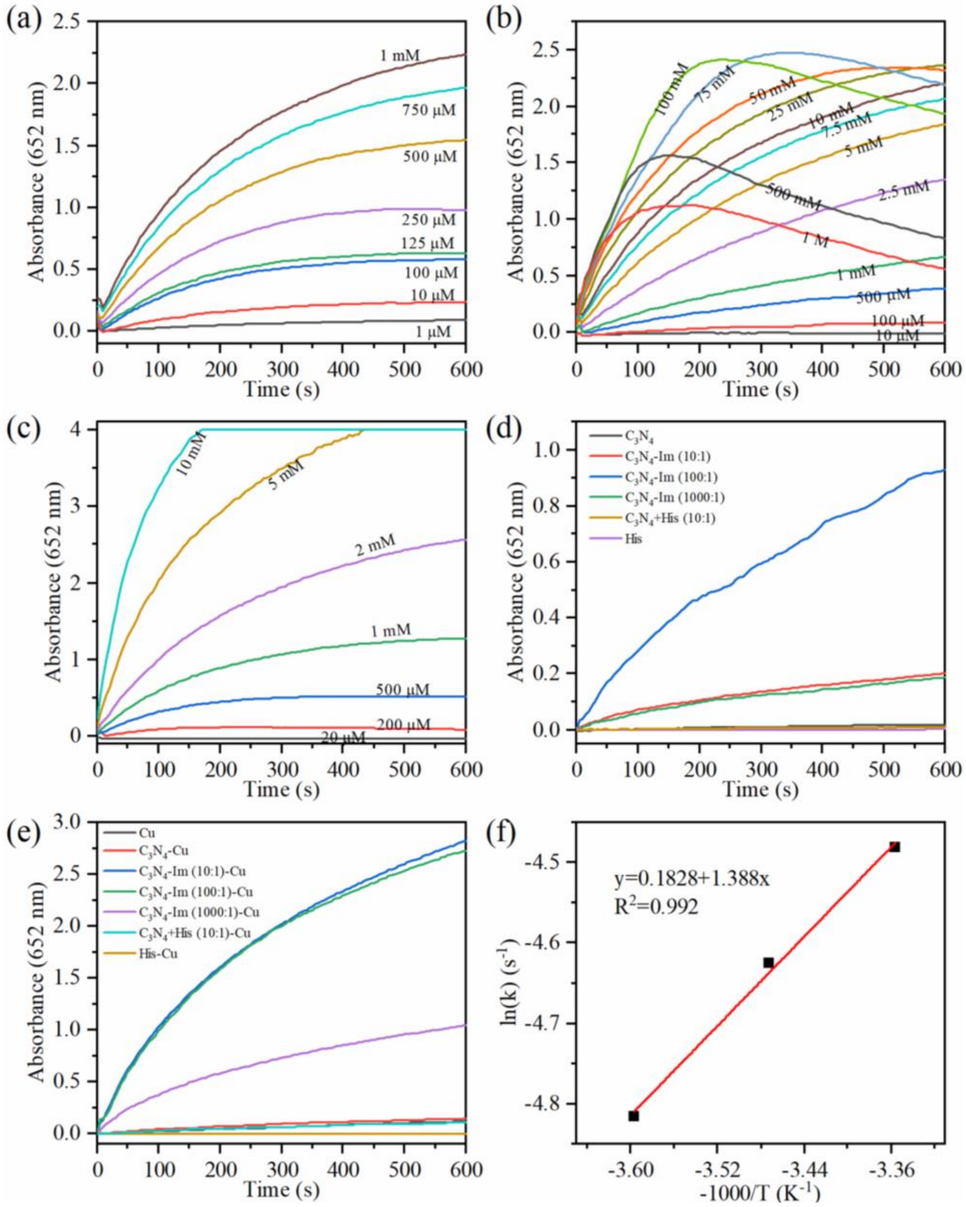

Figure S7. Optimal reaction conditions for peroxidase-like activity of g-C_3N_4-Im-Cu. The critical factors in the test of peroxidase-like activity of g-C_3N_4-Im-Cu were a) copper ion concentration, b) H_2O_2 concentration and c) TMB concentration. d) The time-dependent absorbance changes at 652 nm of TMB solutions in the presence of histidine, g-C_3N_4, and g-C_3N_4-Im of different mass ratios between urea and histidine (10:1; 100:1; 1000:1). e) Repeating the experiment in **Figure S5d** after copper ions coordination. In the d) and e), The mixed solution contains H_2O_2 500 μl (100 mM), HAc/NaAc 1.5 ml (10 mM pH=7), catalyst 500 μL and TMB 500 μL (2 mM). f) The Arrhenius plot for H_2O_2 oxidation catalyzed by g-C_3N_4-Im-Cu, the test curves were calculated at the selected temperatures of 5 ^{o}C, 15 ^{o}C and 25 ^{o}C.

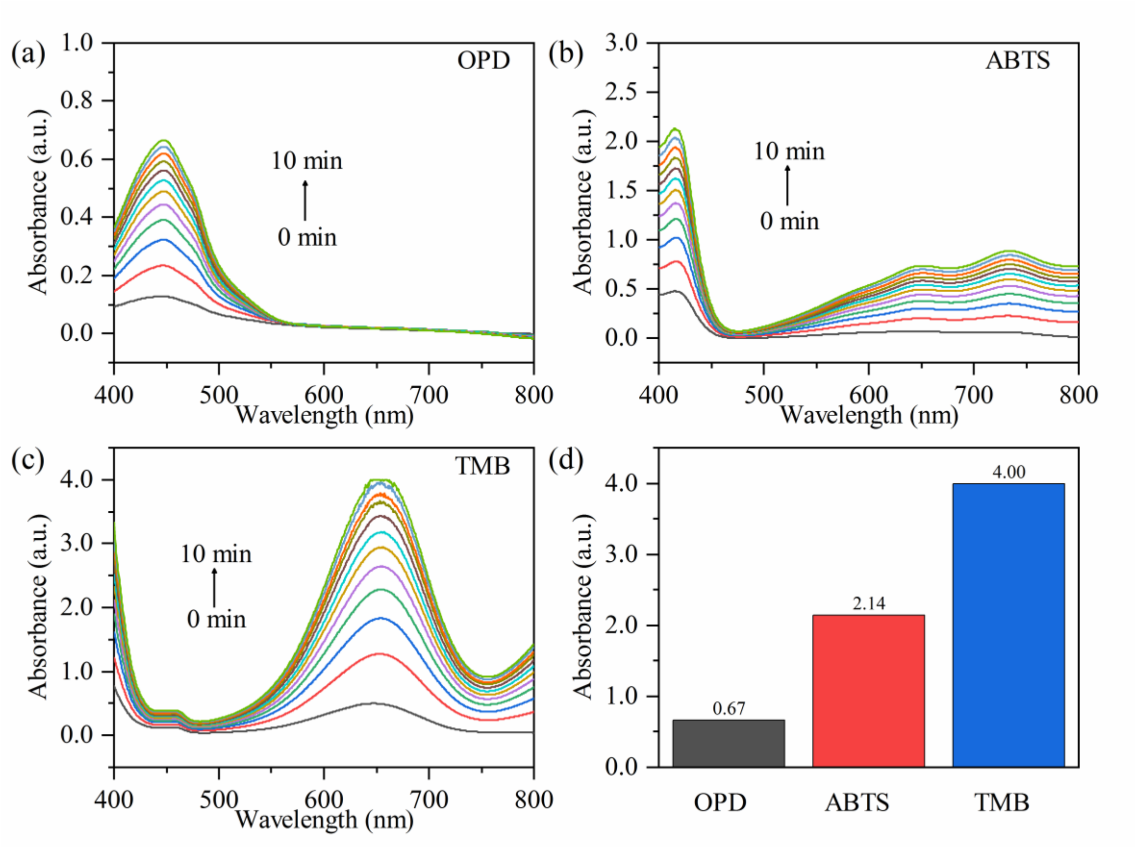

Figure S8. Peroxidase-like activity of g-C_3N_4-Im-Cu with various substrates. UV-vis spectra of H_2O_2 and g-C_3N_4-Im-Cu mixture in the presence of a) OPD, b) ABTS and c) TMB. d) Absorbance value of the absorption peaks of the three peroxidase substrates. The mixture contains H_2O_2 500 μl (100 mM), HAc/NaAc 1.5 ml (10mM pH=7), g-C_3N_4-Im-Cu 500 μL (The concentrations of g-C_3N_4-Im and Cu(II) ion are 0.25 mg/ml and 1 mM respectively) and peroxidase substrates 500 μL (2 mM).

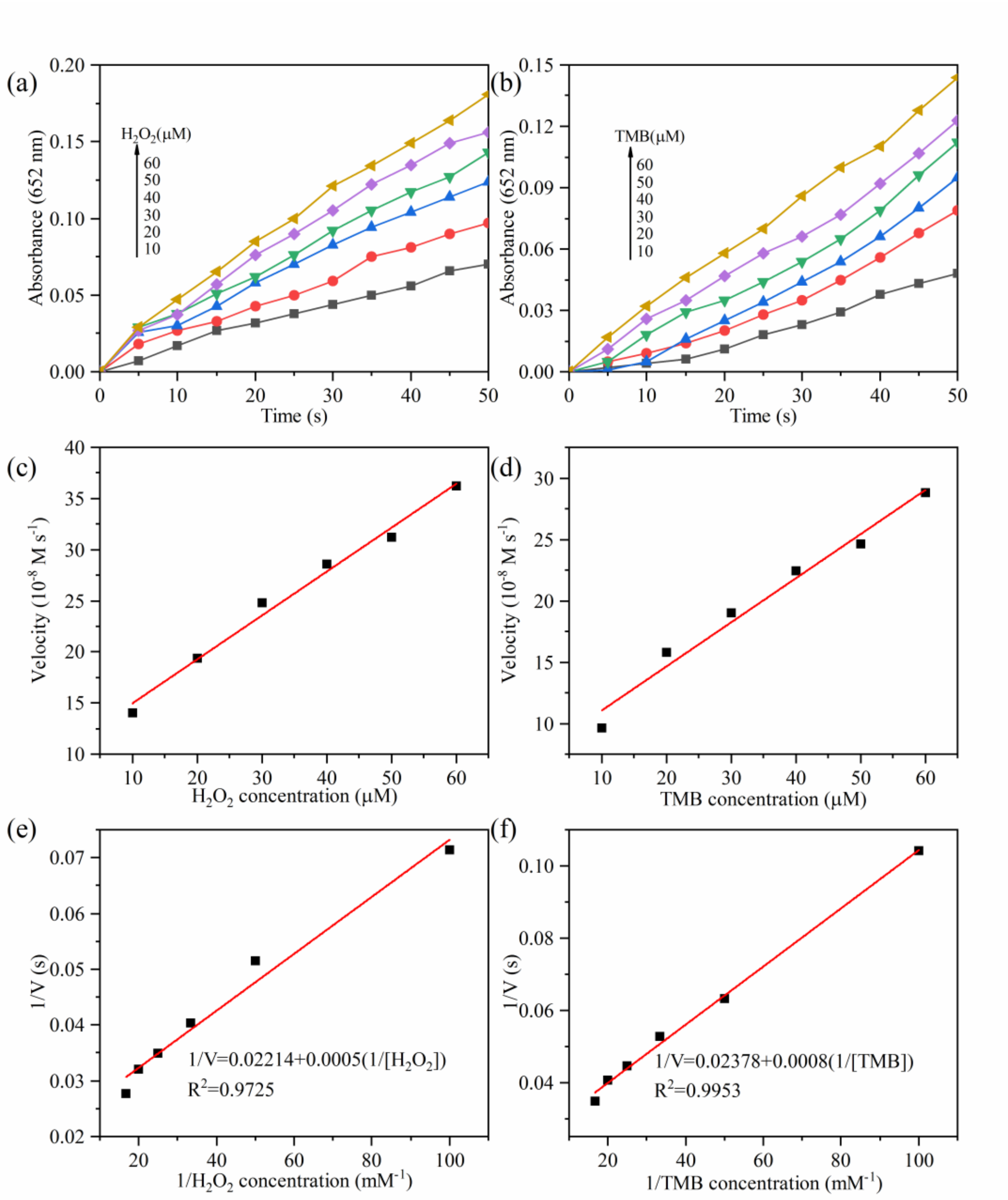

Figure S9. Steady-state kinetic assay of peroxidase-like activity of g-C_3N_4-Im-Cu. a-b) Time-dependent absorbance changes at 652 nm of TMB catalyzed by g-C_3N_4-Im-Cu in the presence of TMB or H_2O_2 with different concentrations. c-d) The velocity (V) of the reaction changes in the presence of TMB or H_2O_2 with different concentrations. e-f) Double reciprocal plots of activity of g-C_3N_4-Im-Cu in the presence of TMB or H_2O_2 with different concentrations. Experiments were carried out in 10 mM acetic acid-sodium acetate buffer (pH=7) using g-C_3N_4-Im-Cu as catalyst at 15 °C. a, c, e) H_2O_2 concentration was fixed at 100 mM and TMB concentration was varied. b, d, f) TMB concentration was fixed at 2 mM and H_2O_2 concentration was varied.

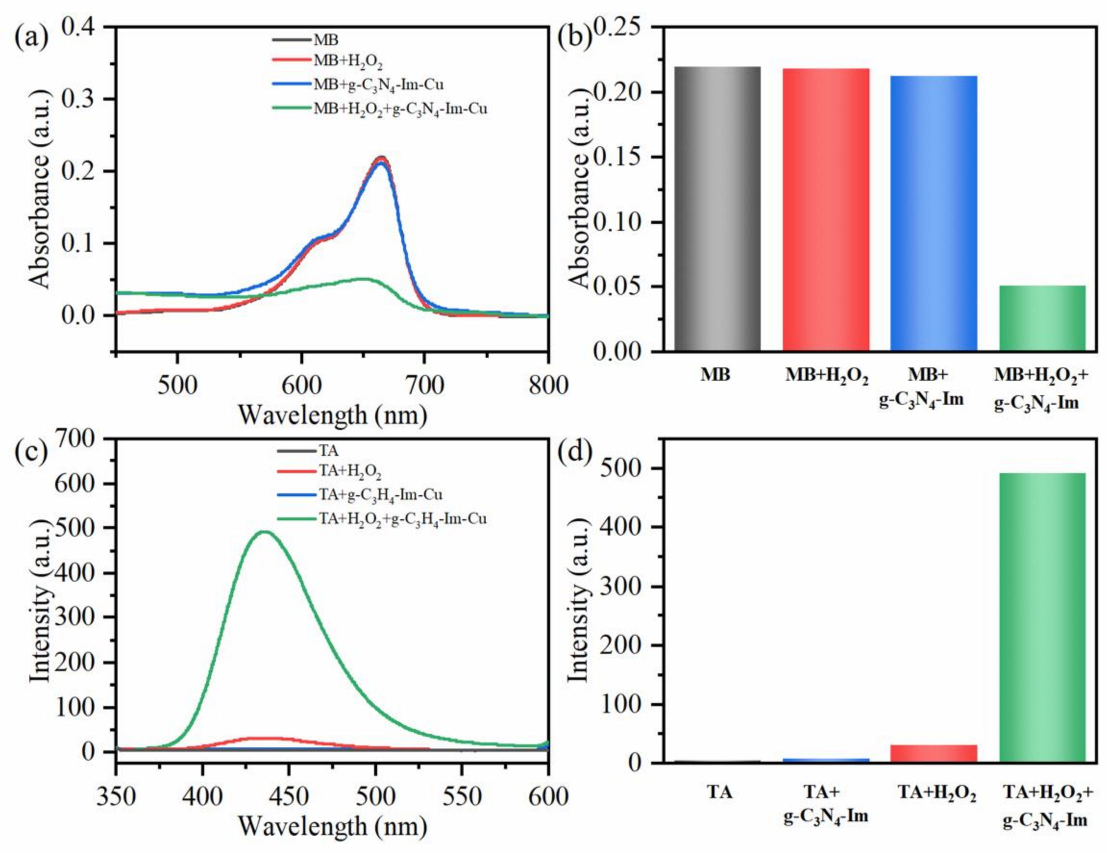

Figure S10. Detecting hydroxyl radical (•OH) with UV-vis and fluorescence spectra. a-b) UV-vis spectra of MB, MB + H_2O_2, MB + g-C_3N_4-Im-Cu, MB + g-C_3N_4-Im-Cu + H_2O_2 after 0.5 h of reaction in acetic acid-sodium acetate buffer solution, respectively. The mixed solution contains H_2O_2 500 µl (100 mM), HAc/NaAc 1.5 ml (10 mM, pH=7), g-C_3N_4-Im-Cu 500 µl and MB 500 µl (2 mM). c-d) Fluorescence spectra of acetic acid-sodium acetate buffer solution include TA, TA + H_2O_2, TA + g-C_3N_4-Im-Cu, TA + H_2O_2 + g-C_3N_4-Im-Cu after 12 h reaction. The mixed solution contains H_2O_2 500 µl (100 mM), HAc/NaAc 1.5 ml (10 mM, pH=7), g-C_3N_4-Im-Cu 500 µl and TA 500 µl (2 mM).

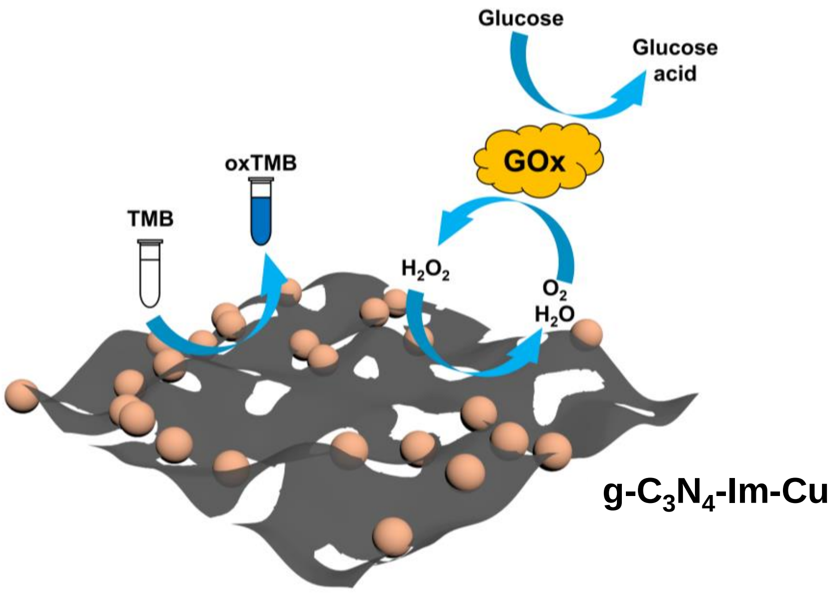

Figure S11. Schematic illustration of glucose detection with glucose oxidase (GOx) and g-C_3N_4-Im-Cu catalyzed reactions.

Table S1. Activation energy calculation results.

T(K)	-1(K^{-1})	k(s^{-1})	Ea (kJ•mol^{-1})	A (s^{-1})
278	-0.0036	0.0081	11.54	1.2
288	-0.00347	0.0098		
298	-0.00336	0.01132		

Table S2. Activation energy for H_2O_2 oxidation catalyzed by different catalysts.

Number	Sample	Ea (kJ•mol^{-1})	Reference
1	ZrO_2	33±1	[s1]
2	TiO_2	34±1	[s2]
3	Y_2O_3	44±5	[s2]
4	MoS_2/GO	24.64	[s3]
5	GO	28.8	[s4]
6	g-C_3N_4-Im-Cu	11.54	This work

Table.S3. Limit of detection (LOD) of various nanozymes for glucose.

No.	Catalysts	Limit of detection (μM)	Reference
1	$Fe_3O_4@CeO_2$	21	[s5]
2	Corrole-Fe_3O_4 nanocomposites	2.46	[s6]
3	Fe_3O_4 NPs	30	[s7]
4	Fe-porphyrin-based covalent organic framework (Fe-COF)	1	[s8]
5	Co_3O_4	0.32	[s9]
6	MoS_2/GO	0.086	[s3]
7	$MoS_2@MgFe_2O_4$	2	[s10]
8	MoO_3/C	10	[s11]
9	VS_2	1.5	[s12]
10	MnO_2 NFs	5	[s13]
11	GOD-GO/MnO_2	17	[s14]
12	Carbon quantum dots (CQDs)	3	[s15]
13	GOx@ZIF-8@Fe-PDA	1.1	[s16]
14	MnO_2	10	[s17]
15	NiFe-LDHNS	50	[s18]
16	multielement-doped carbon dots (ME-CDs)	60	[s19]
17	CoO-OMC nanocomposite	68	[s20]
18	$Fe_3O_4@SiO_2$-NH_2-Au@$Pd_{0.30}$NPs	0.06	[s21]
19	rhodium nanoparticles (RhNPs)	0.75	[s22]
20	Ag@Au core/shell triangular nanoplates	800	[s23]

21	Au-Ni/g-C_3N_4	1.7	[s24]
22	AgNPs/GQDs	0.03	[s25]
23	PtAg-multi-walled carbon nanotubes	600	[s26]
24	g-C_3N_4	0.4	[s27]
25	Fe-g-C_3N_4	0.5	[s28]
26	Cu NPs/g-C_3N_4	0.37	[s29]
27	PdNPs/g-C_3N_4	0.4	[s30]
28	g-C_3N_4-PdNPs	1	[s31]
29	Ru–C_3N_4	0.1	[s32]
30	MnSe-g-C_3N_4	8	[s33]
31	CuPd@H–C_3N_4	0.1	[s34]
32	g-C_3N_4-Im-Cu	0.01	This work

Reference

[s1] Lousada, C. M.; Jonsson, M. Kinetics, mechanism, and activation energy of H_2O_2 decomposition on the surface of ZrO_2. *J. Phys. Chem. C* **2010**, *114* (25), 11202-11208.

[s2] Lousada, C. M.; Johansson, A. J.; Brinck, T.; Jonsson, M. Mechanism of H_2O_2 decomposition on transition metal oxide surfaces. *J. Phys. Chem. C* **2012**, *116* (17), 9533-9543.

[s3] Peng, J.; Weng, J. Enhanced peroxidase-like activity of MoS_2/graphene oxide hybrid with light irradiation for glucose detection. *Biosens.Bioelectron.* **2017**, *89*, 652-658.

[s4] Wang, Z.; Lv, X.; Weng, J. High peroxidase catalytic activity of exfoliated few-layer graphene. *Carbon* **2013**, *62*, 51-60.

[s5] Huang, F.; Wang, J.; Chen, W.; Wan, Y.; Wang, X.; Cai, N.; Liu, J.; Yu, F. Synergistic peroxidase-like activity of CeO_2-coated hollow Fe_3O_4 nanocomposites as an enzymatic mimic for low detection limit of glucose. *Journal of the Taiwan Institute of Chemical Engineers* **2018**, *83*, 40-49.

[s6] Gao, L.; Zhang, L.; Lyu, X.; Lu, G.; Liu, Q. Corrole functionalized iron oxide nanocomposites as enhanced peroxidase mimic and their application in H_2O_2 and glucose colorimetric sensing. *Engineered Science* **2018**, *1* (14), 69-77.

[s7] Chen, M.; Sun, L.; Ding, Y.; Shi, Z.; Liu, Q. N, N′-Di-carboxymethyl perylene diimide functionalized magnetic nanocomposites with enhanced peroxidase-like activity for colorimetric sensing of H_2O_2 and glucose. *New J. Chem* **2017**, *41* (13), 5853-5862.

[s8] Wang, J.; Yang, X.; Wei, T.; Bao, J.; Zhu, Q.; Dai, Z. Fe-Porphyrin-based covalent organic framework as a novel peroxidase mimic for a one-pot glucose colorimetric assay. *ACS Appl. Bio Mater.* **2018**, *1* (2), 382-388.

[s9] Lu, J.; Zhang, H.; Li, S.; Guo, S.; Shen, L.; Zhou, T.; Zhong, H.; Wu, L.; Meng, Q.; Zhang, Y. Oxygen-vacancy-enhanced peroxidase-like activity of reduced Co_3O_4 nanocomposites for the colorimetric detection of H_2O_2 and glucose. *Inorg. Chem.* **2020**, *59* (5), 3152-3159.

[s10] Zhang, Y.; Zhou, Z.; Wen, F.; Tan, J.; Peng, T.; Luo, B.; Wang, H.; Yin, S. A flower-like MoS_2-decorated $MgFe_2O_4$ nanocomposite: Mimicking peroxidase and colorimetric detection of H_2O_2 and glucose. *Sens. Actuators B Chem.* **2018**, *275*, 155-162.

[s11] Ren, H.; Yan, L.; Liu, M.; Wang, Y.; Liu, X.; Liu, C.; Liu, K.; Zeng, L.; Liu, A. Green tide biomass templated synthesis of molybdenum oxide nanorods supported on carbon as efficient nanozyme for sensitive glucose

colorimetric assay. *Sens. Actuators B Chem.* **2019**, *296*, 126517.
[s12] Huang, L.; Zhu, W.; Zhang, W.; Chen, K.; Wang, J.; Wang, R.; Yang, Q.; Hu, N.; Suo, Y.; Wang, J. Layered vanadium (IV) disulfide nanosheets as a peroxidase-like nanozyme for colorimetric detection of glucose. *Microchimica Acta* **2018**, *185* (1), 1-8.
[s13] Han, L.; Zhang, H.; Chen, D.; Li, F. Protein-directed metal oxide nanoflakes with tandem enzyme-like characteristics: colorimetric glucose sensing based on one-pot enzyme-free cascade catalysis. *Adv. Funct. Mater.* **2018**, *28* (17), 1800018.
[s14] Lee, P.-C.; Li, N.-S.; Hsu, Y.-P.; Peng, C.; Yang, H.-W. Direct glucose detection in whole blood by colorimetric assay based on glucose oxidase-conjugated graphene oxide/MnO_2 nanozymes. *Analyst* **2019**, *144* (9), 3038-3044.
[s15] Zhong, Q.; Chen, Y.; Qin, X.; Wang, Y.; Yuan, C.; Xu, Y. Colorimetric enzymatic determination of glucose based on etching of gold nanorods by iodine and using carbon quantum dots as peroxidase mimics. *Microchimica Acta* **2019**, *186* (3), 161.
[s16] Zhao, Z.; Lin, T.; Liu, W.; Hou, L.; Ye, F.; Zhao, S. Colorimetric detection of blood glucose based on GO_x@ZIF-8@Fe-polydopamine cascade reaction. *Spectrochim. Acta A Mol. Biomol.* **2019**, *219*, 240-247.
[s17] Zhang, J.; Dai, X.; Song, Z.-L.; Han, R.; Ma, L.; Fan, G.-C.; Luo, X. One-pot enzyme-and indicator-free colorimetric sensing of glucose based on MnO_2 nano-oxidizer. *Sens. Actuators B Chem.* **2020**, *304*, 127304.
[s18] Zhan, T.; Kang, J.; Li, X.; Pan, L.; Li, G.; Hou, W. NiFe layered double hydroxide nanosheets as an efficiently mimic enzyme for colorimetric determination of glucose and H_2O_2. *Sens. Actuators B Chem.* **2018**, *255*, 2635-2642.
[s19] Wang, B.; Liu, F.; Wu, Y.; Chen, Y.; Weng, B.; Li, C. M. Synthesis of catalytically active multielement-doped carbon dots and application for colorimetric detection of glucose. *Sens. Actuators B Chem.* **2018**, *255*, 2601-2607.
[s20] Guo, Y.; Yan, L.; Zhang, R.; Ren, H.; Liu, A. CoO-supported ordered mesoporous carbon nanocomposite based nanozyme with peroxidase-like activity for colorimetric detection of glucose. *Process Biochem* **2019**, *81*, 92-98.
[s21] Adeniyi, O.; Sicwetsha, S.; Mashazi, P. Nanomagnet-silica nanoparticles decorated with Au@Pd for enhanced peroxidase-like activity and colorimetric glucose sensing. *ACS Appl. Mater. Interfaces* **2019**, *12* (2), 1973-1987.
[s22] Choleva, T. G.; Gatselou, V. A.; Tsogas, G. Z.; Giokas, D. L. Intrinsic peroxidase-like activity of rhodium nanoparticles, and their application to the colorimetric determination of hydrogen peroxide and glucose. *Microchimica Acta* **2018**, *185* (1), 1-9.

[s23] Liu, A.; Li, M.; Wang, J.; Feng, F.; Zhang, Y.; Qiu, Z.; Chen, Y.; Meteku, B. E.; Wen, C.; Yan, Z. Ag@Au core/shell triangular nanoplates with dual enzyme-like properties for the colorimetric sensing of glucose. *Chin Chem Lett* **2020**, *31* (5), 1133-1136.
[s24] Darabdhara, G.; Bordoloi, J.; Manna, P.; Das, M. R. Biocompatible bimetallic Au-Ni doped graphitic carbon nitride sheets: a novel peroxidase-mimicking artificial enzyme for rapid and highly sensitive colorimetric detection of glucose. *Sens. Actuators B Chem* **2019**, *285*, 277-290.
[s25] Nguyen, N. D.; Van Nguyen, T.; Chu, A. D.; Tran, H. V.; Tran, L. T.; Huynh, C. D. A label-free colorimetric sensor based on silver nanoparticles directed to hydrogen peroxide and glucose. *Arab. J. Chem.* **2018**, *11* (7), 1134-1143.
[s26] Fu, S.; Zhu, C.; Song, J.; Engelhard, M.; Xia, H.; Du, D.; Lin, Y. PdCuPt nanocrystals with multibranches for enzyme-free glucose detection. *ACS Appl. Mater. Interfaces* **2016**, *8* (34), 22196-22200.
[s27] Liu, J.-W.; Luo, Y.; Wang, Y.-M.; Duan, L.-Y.; Jiang, J.-H.; Yu, R.-Q. Graphitic carbon nitride nanosheets-based ratiometric fluorescent probe for highly sensitive detection of H_2O_2 and glucose. *ACS Appl. Mater. Interfaces* **2016**, *8* (49), 33439-33445.
[s28] Peng, Y.; Yu, X.; Yin, W.; Dong, W.; Peng, J.; Wang, T. Colorimetric assay using mesoporous Fe-doped graphitic carbon nitride as a peroxidase mimetic for the determination of hydrogen peroxide and glucose. *ACS Appl. Bio Mater.* **2019**, *3* (1), 59-67. DOI: 10.1021/acsabm.9b00883
[s29] Wang, N.; Han, Z.; Fan, H.; Ai, S. Copper nanoparticles modified graphitic carbon nitride nanosheets as a peroxidase mimetic for glucose detection. *RSC Adv.* **2015**, *5* (111), 91302-91307.
[s30] Zhang, W.; Li, X.; Xu, X.; He, Y.; Qiu, F.; Pan, J.; Niu, X. Pd nanoparticle-decorated graphitic C_3N_4 nanosheets with bifunctional peroxidase mimicking and ON-OFF fluorescence enable naked-eye and fluorescent dual-readout sensing of glucose. *J Mater Chem B* **2019**, *7* (2), 233-239.
[s31] Jin, X.; Zhong, Y.; Chen, L.; Xu, L.; Wu, Y.; Fu, F. A Palladium-Doped Graphitic Carbon Nitride Nanosheet with High Peroxidase-Like Activity: Preparation, Characterization, and Application in Glucose Detection. *Part Part Syst Charact* **2018**, *35* (3), 1700359.
[s32] Deng, W.; Peng, Y.; Yang, H.; Tan, Y.; Ma, M.; Xie, Q.; Chen, S. Ruthenium ion-complexed carbon nitride nanosheets with peroxidase-like activity as a ratiometric fluorescence probe for the detection of hydrogen peroxide and glucose. *ACS Appl. Mater. Interfaces* **2019**, *11* (32), 29072-29077.
[s33] Qiao, F.; Qi, Q.; Wang, Z.; Xu, K.; Ai, S. MnSe-loaded g-C_3N_4 nanocomposite with synergistic peroxidase-like catalysis: synthesis and application toward colorimetric biosensing of H_2O_2 and glucose. *Sens. Actuators B Chem.* **2016**, *229*, 379-386.

[s34] Tang, W.; An, Y.; Chen, J.; Row, K. H. Multienzyme mimetic activities of holey CuPd@H-C_3N_4 for visual colorimetric and ultrasensitive fluorometric discriminative detection of glutathione and glucose in physiological fluids. *Talanta* **2022**, 123221.
